# Bacterial Cellulose—Graphene Based Nanocomposites

**DOI:** 10.3390/ijms21186532

**Published:** 2020-09-07

**Authors:** Omar P. Troncoso, Fernando G. Torres

**Affiliations:** Department of Mechanical Engineering, Pontificia Universidad Católica del Perú, Lima 15088, Peru; troncoso.op@pucp.pe

**Keywords:** bacterial cellulose, graphene, supercapacitors, biomedical applications, water purification

## Abstract

Bacterial cellulose (BC) and graphene are materials that have attracted the attention of researchers due to their outstanding properties. BC is a nanostructured 3D network of pure and highly crystalline cellulose nanofibres that can act as a host matrix for the incorporation of other nano-sized materials. Graphene features high mechanical properties, thermal and electric conductivity and specific surface area. In this paper we review the most recent studies regarding the development of novel BC-graphene nanocomposites that take advantage of the exceptional properties of BC and graphene. The most important applications of these novel BC-graphene nanocomposites include the development of novel electric conductive materials and energy storage devices, the preparation of aerogels and membranes with very high specific area as sorbent materials for the removal of oil and metal ions from water and a variety of biomedical applications, such as tissue engineering and drug delivery. The main properties of these BC-graphene nanocomposites associated with these applications, such as electric conductivity, biocompatibility and specific surface area, are systematically presented together with the processing routes used to fabricate such nanocomposites.

## 1. Introduction

Cellulose is an abundant inexpensive biopolymer, traditionally extracted from plants and biomass waste. The cellulose synthetized by plants is usually branched with hemicellulose and lignin. It has to undergo chemical process to obtain the pure cellulose [1]. Some acetic acid bacteria can synthetize cellulose extracellularly. This bacterial cellulose (BC) is formed only by glucose monomers without hemicellulose or lignin. Glucose chain protofibrils are secreted through bacteria cell wall and aggregate together forming nanofibrils cellulose ribbons [2]. These cellulose ribbons aggregate themselves to form a coherent 3D cellulose network. As a result, a pellicle with high surface area and porosity is formed. This cellulose nanofibre network has high mechanical strength, high crystallinity, high degree of polymerization and high water holding capacity. Table 1 summarizes some of the most important properties of BC network.

Pure BC features uncontrolled hydrolysis rate and low biological activity. BC-based nanocomposites have been developed to improve the capabilities of pure BC. BC nanocomposites have been used for the development of high strength paper and polymeric films, high strength membranes and as a reinforcing agent for polymer nanocomposites [3]. In addition, due to its non-toxicity and biocompatibility, BC-based materials have also been used for biomedical applications such as biological implants, artificial blood vessels for microsurgery, scaffolds for tissue engineering and wound dressing patches, among others [4].

Graphene is another nanomaterial that has attracted extensive interest due to its outstanding physicochemical properties, including high Young’s modulus, high electrical and thermal conductivity, quick charge carrier mobility and large specific surface area (Table 1). Graphene is the first truly two dimensional (2D) crystal ever discovered [5,6]. A graphene crystal is composed of six-atom rings in a honeycombed network with one-atom thickness, forming a free-standing 2-D crystal that can function as a planar aromatic macromolecule [7]. Each carbon atom is bonded to three other carbon atoms, forming three σ-bonds via three sp2 hybrid orbitals. The other p-orbital form a conjugated system with other contiguous carbon atoms. As a result, the carbon skeleton of graphene is made of σ-bonds with paired electron clouds above and below the skeleton. Graphene can be transformed into spherical structures (0-D fullerenes), tubular structures (one-dimensional carbon nanotubes, 1-D CNTs) or layered structures (3-D graphite) [8]. Graphene, graphene oxide (GO) and reduced graphene oxide (RGO) have found a variety of applications in quantum physics, nanoelectronics, catalysis, nanocomposites and sensor technology [9,10,11,12,13].

Pristine graphene was first obtained in 2004, by exfoliating highly oriented pyrolytic graphite [5]. Other processing techniques have been developed in order to increase the productivity and reduce the cost of graphene. These techniques have allowed for the fabrication of a variety of graphene family nanomaterials (GFNs). Among them, graphene oxide (GO) and reduced graphene oxide (RGO) are the most commonly used.

GO is usually obtained by chemical exfoliation following the Hummers method [14]. Briefly, graphite and NaNO_3_ are added to concentrated sulfuric acid, followed by KMnO_4_ as the oxidizing agent to oxidize the graphite. Then, 30% H_2_O_2_ is added to reduce the oxidizing agent and obtain GO. However, GO has not the same properties as graphene. GO is hydrophilic and electrically insulating. During the chemical exfoliation of graphite, a significant fraction of the sp2 carbon network is bonded with oxygen-containing hydrophilic groups. This causes the disruption of the sp2 bonding network in its carbon basal plane [15,16,17]. Thus, for some applications the GO has to be reduced in order to restore some of the properties of pristine graphene. There are a variety of processing routes to reduce GO, including chemical reduction, photocatalytic reduction, electrochemical reduction and thermal reduction [18,19,20,21].

BC is a biopolymer that forms nanostructured 3D network with outstanding mechanical properties. This BC network can be used as matrix for the development of novel nanocomposites with tailored properties. On the other hand, GFNs features remarkable properties such as thermal and electronic conductivity as well as high mechanical properties. This review offers an overview of the most recent BC-graphene nanocomposites developed to take advantage of the exceptional properties of BC and GFNs. Three main applications were identified for these BC-GFN nanocomposites, including the development of novel electric conductive materials and energy storage devices, the preparation of aerogels and membranes with very high specific area as sorbent materials for the removal of oil and metal ions from water and a variety of biomedical applications, such as tissue engineering and drug delivery. The main properties of these BC-GFN nanocomposites associated with these applications, such as electric conductivity, biocompatibility and specific surface area, are systematically presented together with the processing routes used to fabricate such nanocomposites. This review can be used as a guide to support researchers for the development of novel graphene- and cellulose- based materials for different applications including green electronic devices, water purification systems and biomedical materials, among other applications that have not been considered yet.

## 2. Processing Routes

Bacterial cellulose can be synthetized by different bacteria, including Gram negative bacteria such as *Gluconacetobacter xylinus* [23,28], *Agrobacterium* [29], *Rhizobium* [30] and Gram positive bacteria such as *Sarcina* [31]. The preparation of BC-based nanocomposites includes a culture step for bacterial growth. Different culture media have been used to improve the cellulose synthesis.

Mohammadkazemi et al. [32] compared the effects of using different carbon sources and grow media. The carbon sources they used were syrup, glucose, mannitol and sucrose while the grow media were the Hestrin–Schramm, Yamanaka and Zhou media. They found that mannitol lead to the highest yield and that the crystallinity of the BC in presence of the Hestrin–Schramm medium had higher values than those from other media. The culture of bacteria is normally carried out in static conditions but some studies have used agitated conditions. When BC is cultivated in static conditions, a cellulose pellicle is formed at the medium surface exposed to air, while in agitated condition; the spherical or asterisk-like shape cellulose particles are obtained [33].

These pellicles are formed by a cellulose network made from fibres having ~100 nm in diameter [4]. When combining BC with another nano-sized element the developed material can be regarded as a nano-nano composite. Many different processing routes have been reported for the preparation of BC-based nano-nano composites, including BC-Graphene nanocomposites [3]. When preparing BC-Graphene nanocomposites, there are two important aspects that need to be considered. Firstly, we need to consider if the pristine BC network structure has to be preserved. When the BC network remains undisturbed, the resulting nanocomposites take advantage of the intrinsic mechanical robustness of pristine BC. However, the incorporation of a second phase into this cellulose network can be a challenging task and, thus, the disintegration of BC has also been used as a strategy to combine BC and graphene.

The second aspect to be considered is how the graphene platelets are incorporated into the BC network. Graphene can be incorporated after the cellulose fibres have been synthetized or during the cellulose synthesis. For instance, one common way of incorporating graphene is by immersing a pristine BC pellicle in graphene dispersion [34]. A different approach consists in modifying the culture medium of bacteria adding a graphene dispersion in order to allow the synthesis of cellulose in the presence of graphene platelets. As a result, a BC pellicle containing graphene platelets is obtained.

**Figure 1:**
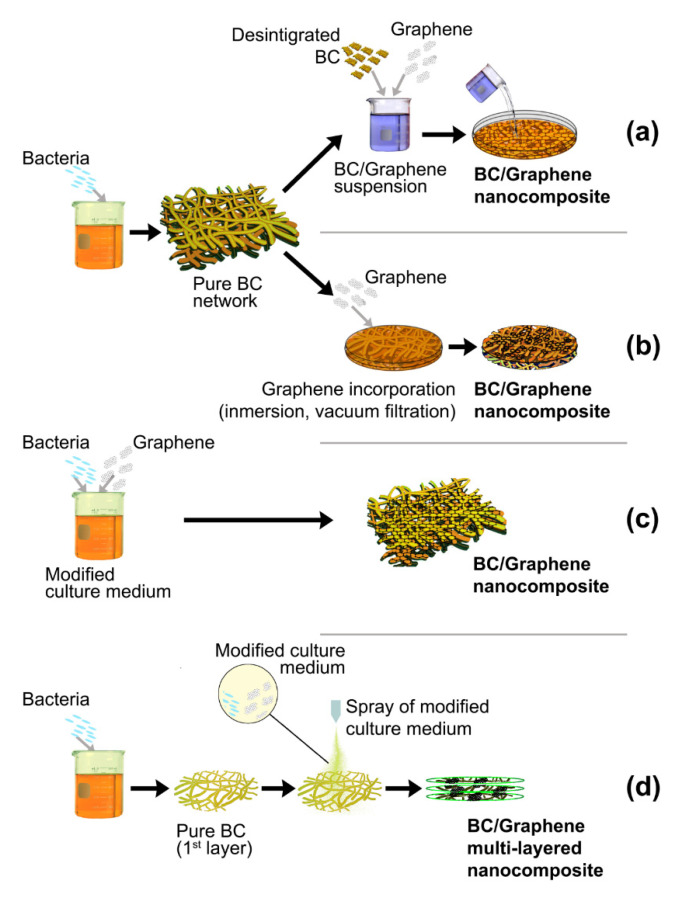
Processing routes reported for the preparation of BC- graphene oxide (GO) nanocomposites. In the first route, the pure BC membrane is disintegrated and a GO-BC suspension is prepared in order to prepare a composite BC-GO film (**a**). In the second route, GO is incorporated into the preserved BC network (**b**). In the third processing route, the BC growing medium is modified by the addition of a GO suspension and the BC network is synthetized in the presence of GO (**c**). For the fourth processing route a conventional growing medium is used to grow a first BC pellicle. Then, a modified growing medium is prepared adding a GO suspension. This modified medium is sprayed onto the first BC pellicle forming a thin layer of culture medium on which new BC nanofibres are synthetized in presence of GO (**d**).

Figure 1 shows three of the most common processing routes used for the preparation of BC-GO nanocomposites. In the first and second routes, BC synthesis is carried out in an unmodified medium and a BC pellicle is obtained. Then, this BC pellicle can be either disintegrated (Figure 1a) or preserved (Figure 1b). The BC is disintegrated by means of a homogenizer or pulping equipment and a BC slurry is obtained [35,36,37,38,39,40]. This BC slurry is then combined with a GO dispersion. Usually, a sonication or some other process is used to achieve a homogenous dispersion. Then, the BC-GO dispersion is dried, pressed or filtered to form a solid film. When the network structure of BC is preserved (Figure 1b) GO is incorporated by an impregnation step and, then, the BC pellicle with the GO incorporated is dried or pressed. Alternatively, a GO dispersion is poured on a BC pellicle and vacuum-dried to incorporate BC and obtain a film in a single step.

For instance, Fang et al. [35] prepared BC-GO membranes following the first route (Figure 1a) for selective ion permeation. They dried and disintegrated the pristine BC pellicle. Then, they dispersed it in formamide with sonication for 2 h at 400 W to prepare stable BC dispersion with concentration of 1 mg mL^−1^. 15 mL of the BC dispersion was mixed with 5 mL of a GO dispersion and sonicated for 10 min. Then the mixture was vacuum filtrated to prepare BC and BC/GO membranes.

The disintegrated BC network can easily undergo chemical treatment. Liu et al. [41] prepared a BC-GO cross-linked nanocomposite by a one-step esterification process. They prepared a dispersion of freshly exfoliated GO sheets and disintegrated BC pellicles in anhydrous N,N-dimethyl formamide (DMF). Dicy clohexyl carbodiimide (DCC) was added as a dehydration reagent. The esterification between carboxyl groups of GO and hydroxyl groups of BC was conducted under nitrogen atmosphere at 70 °C 1 for 48 h to create the cross-linking BC/GO networks. Then, the reaction mixture was filtered through a nylon membrane, rinsed with ethanol and deionized water. The film was finally air-dried at room temperature.

The third route shown in Figure 1c is performed by modifying the culture medium of BC by adding a BC suspension in it. The bacteria synthetize the BC fibres in the presence of the GO platelets and a BC pellicle obtained has GO platelets incorporated in the BC network without any further treatment [42,43,44,45]. Compared with the first route (Figure 1a), when using undisturbed BC membranes there is no need of energy-intensive disintegration process or BC solubilisation in organic solvents/ionic liquids [46], which could have issues related to environmental toxicity and biocompatibility. In addition, the disintegration of BC reduces the mechanical strength and damages the integrity of the BC network. However, incorporating GO platelets into the 3D BC network could be a challenging task, especially when the sample thickness is large (ca. 3 mm) [47].

In order to overcome the difficulties of incorporating graphene into the BC network, a novel layer-by-layer self-assembly route (Figure 1d) has been proposed [47,48,49,50]. First, a conventional growing medium is used to grow a first BC pellicle. A modified growing medium is prepared adding a graphene suspension. Then, this modified medium is sprayed onto the first BC pellicle forming a thin layer of culture medium on which new BC nanofibres are synthetized in presence of graphene platelets. This process is repeated several times until the desired thickness is achieved. This layer-by-layer method results in the formation of mechanically strong and thick three dimensional BC-GO network [50].

Some modifications have been proposed to the aforementioned routes when a third phase is incorporated to form novel BC-GO hybrid nanomaterials. For instance, Chen et al. [51] used an in-situ synthesis of PEDOT (poly-3,4-ethylenedioxythiophene) to prepared BC-GO-PEDOT conductive films. They immersed a dry BC pellicle into an 3,4-Ethylenedioxythiophene (EDOT)- FeCl3 ethanol solution). During the ethanol evaporation, the EDOT monomer started to form a PEDOT shell on the surface of the BC nanofibres. Then, a GO solution was dropped onto the BC-PEDOT film by spin−spray method. Wan et al. [52] and Luo et al. [48] have also made a hybrid nanomaterial using a conductive polymer. They first prepared a BC-GO nanocomposite using the layer-by-layer self-assembly route. Then, they immersed this BC-GO nanocomposite in a HCl aqueous solution containing aniline monomer. The solution was vigorously agitated for 24 h. An ammonium persulfate solution was added drop- wise to promote the in-situ oxidative polymerization of aniline on the surface of the BC-GO nanocomposite to obtain a BC-GO-PANI hybrid. Other conductive polymers, such as polypyrrole and polyethylenimine have also been in-situ polymerized on BC-GO nanocomposites following similar processing routes [53,54].

The BC-GO nanocomposites can be produced as hydrogels, films or aerogels. BC is synthetized by bacteria as a network of nanocellulose fibres that hold a large amount of water. BC-graphene hydrogels can be obtained modifying the bacteria culture medium without further processing. When a membrane or film is needed, water is extracted from the BC network by air-drying, hot- and cold-pressing or vacuum-filtration. BC-GO aerogel can be prepared from BC-based hydrogels by freeze-drying or by a supercritical CO_2_ method [55,56,57].

## 3. Applications of Bacterial Cellulose-Graphene Nanocomposites

### 3.1. Electronic and Energy Storage Devices

Single graphene layers have the highest known electrical conductivity [58]. Many researchers have used graphene as filler to prepare conductive polymer matrix composites [59,60,61], in spite of the fact that polymers are electric insulators. It has been reported that the incorporation of graphene can lead to a percolation behaviour at very low volume fractions and can increase the conductivity of the resulting composites by several orders of magnitude [62,63,64]. It has been reported that polymeric composites reinforced with carbon nanofillers, such as carbon nanotubes and graphene, feature an electric tunnelling effect. If the polymer layer between carbon intrusions is thing enough (~1–2 nm) electrons can cross and current can flow between both inclusions [65,66,67].

RGO has been incorporated to a BC matrix in order to prepare conductive paper and films [52,68,69,70,71]. Ccorahua et al. [70] reported the incorporation of graphene into a pristine BC network by vacuum filtration. The dependence of the electric conductivity on the amount of graphene incorporated suggested the existence of a critical content of RGO that boosted the conductivity of the BC-RGO nanocomposite. The conductivity of BC-RGO was dependent on the GO content. At an RGO content of 20% the conductivity was 1 S∙m^−1^. At an RGO content of 30%, the conductivity was 12 S∙m^−1^. Table 2 shows the electric conductivity of several BC-graphene composites reported in the literature.

These conducting BC-graphene based materials can potentially be used in the assembly of novel energy storage devices. Supercapacitors are among the most promising energy storage devices and are capable of managing high power rates compared to batteries. However, they are not able to store the same amount of charge as batteries [72]. Supercapacitors are, thus, suitable for applications in which power burst are needed but storage capacity is not required. The advantages of supercapacitors include their ability to charge and discharge in an extremely short period of time (seconds) and their long cycle life. There are different types of supercapacitors. Among them, the electrochemical double-layer capacitors (EDLCs) are the most promising type of capacitor to be developed using BC-graphene nanocomposites.

**Figure 2:**
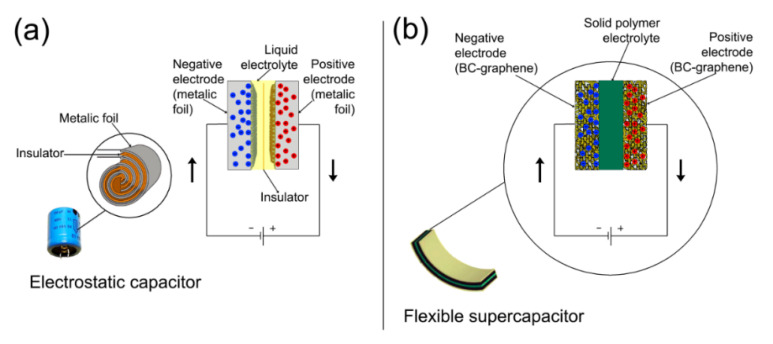
Schematic representation of an electronic capacitor (**a**) and a flexible electrochemical double-layer capacitor (EDLC) (**b**).

In a traditional electrostatic capacitor (Figure 2a), energy is stored via an electrostatic field generated from the removal of charge carriers (i.e., electrons) from one metal plate (i.e., electrode), then depositing them on another [73]. Their charge mechanism does not involve irreversible chemical reactions (there is no Faradic process). Rather, the charges are physically attached on the surface of the electrodes in an electric double layer [74]. EDLCs work much in the same way as electrostatic capacitors (Figure 2b) but using electrodes with a very high surface area. As the capacitance is proportional to the electrode area, EDLCs achieve very high capacitance values (kilo-Farads) compared to electrostatic capacitors (mili- and micro-Farads) [74].

The electrodes of conventional EDLCs are formed by a layer of activated carbon paste deposited onto a metallic current collector. A metallic casting encases the electrodes immersed in a liquid electrolyte (Figure 2a). However, the large micro/macropores within activated carbon is disadvantageous for the adsorption of the electrolyte on the surface of electrodes, deteriorating the function of EDLCs. In addition, for some emerging applications such as wearable electronics, electronic newspapers and paper-like mobile phones [75], conventional EDLCs are heavy and are incompatible with large strains.

Freestanding conductive films are considered to be one potential solution to address the challenge of producing flexible capacitors. Carbon-based paper-like materials, such as RGO paper and carbon nanotube (CNT) paper are potential candidates to be used as flexible electrodes because of high their conductivity and large specific surface area [61]. However, the small mass loading that can be achieved limits their potentials. An alternative solution has been found incorporating graphene on a flexible, porous and light-weight BC substrate. The BC substrate can provide three-dimensional (3D) porous network structure and therefore enable high mass loading. The excellent mechanical properties of BC [76] provide the BC-based electrodes with high mechanical integrity upon bending or folding. Figure 2b shows a schematic representation of an all-Solid-State flexible supercapacitor in which BC-graphene nanocomposite films are used as electrodes.

Several works report the use of BC-graphene nanocomposite films for the production of flexible and highly-efficient supercapacitor electrodes. Table 3 shows that these nanocomposites films exhibited high electrical conductivity (171–1660 S m^−1^) and large specific capacitance (160–556 F g^−1^), compared to activated carbon-based electrodes, along with high cycling stability (86%–100% retention).

Batteries are other energy storage devices that can also been constructed using bacterial cellulose-graphene based nanocomposites. For instance, Shen et al. [40] used pyrolized BC to prepare a nanocomposite with GO for the development of a new Li–S battery. They used the pyrolize BC/GO nanocomposite as a membrane separator placed between the anode and the cathode of the battery. The 3D structure of this BC/GO nanocomposite worked as an effective barrier to retard polysulfide diffusion during the charge/discharge process to enhance the cyclic stability of the Li–S battery. Such Li-S batteries exhibited a specific capacity of nearly 600 mAh.g^−1^ and only 0.055% capacity decay per cycle over 200 cycles. Zhang et al. [78] also used BC-GO membranes as separators for redox flow batteries. They found that the batteries assembled with BC-GO separators had a charge-discharge profile similar to that of the batteries equipped with commercial Nafion 212 membranes and achieve a stable cycling performance with a Coulombic efficiency of ∼98%.

BC-graphene based nanocomposites have also been used to produce other electronic devices such as sensors and actuators. The amperometric response of BC-GO based nanocomposite films has been used to fabricate sensors. Zhang et al. [79] prepared a modified glassy carbon electrode by drop-casting a BC-GO suspension onto the surface of glassy carbon. This modified glassy carbon electrode was used to determine nitrite concentration in water, in a wide linear range of 0.5 to 4590 μM with detection limit and sensitivity of 0.2 μM and 527.35 μA μM^−1^ × cm^−2^, respectively. Lv et al. [80] used the layer-by-layer processing route (Figure 1d) to incorporate nitrogen doped graphene oxide quantum dots (N-GOQDs) in a BC network. This BC/N-GOQD nanocomposite were used for the detection of iron ions in aqueous solutions. Their results showed that the blue-emitting BC/N-GOQD fluorescent probes exhibited a sensitive response to Fe^3+^ within a concentration range of 0.5–650 μM with a lower limit detection of 69 nM. Hosseini et al. [81] prepared a BC/RGO conductive aerogel by a supercritical CO_2_ (ScCO_2_) drying method. They characterized the mechanical and electrical properties of the BC/RGO aerogels and found a gauge factor (ratio between the relative change in electrical resistance to the mechanical strain). This suggests that such a BC/RGO aerogel can effectively be used as a strain sensor.

Kim et al. [82] prepared a bioelectronic soft actuator using 2,2,6,6-tetramethylpiperidine-1-oxyl radical-oxidized bacterial cellulose (TEMPO-BC) and graphene. The top and bottom surface of the TEMPO-BC/graphene film was coated with a conducting electrode made from poly(3,4-ethylenedioxythiophene) polystyrene sulfonate (PEDOT:PSS). The results showed that under sinusoidal excitation of 1.0 V at 0.1 Hz, the tip displacement of the TEMPO-BC/graphene actuator reached peak tip displacements of ±2.5 and ±4 mm, while the displacement of the TEMPO-BC actuators reached a peak of ±1.2 mm. The unique TEMPO-BC/graphene actuator showed large static deformation without apparent back-relaxation, much faster response time and highly durable harmonic actuation compared with other biopolymer actuators.

### 3.2. Sorbent Nanocomposites for Water Purification

BC-graphene nanocomposites have also been used in the construction of devices for the purification of water, specifically for two types of applications—water-oil separation and metal ions removal. BC-graphene aerogels have been developed as efficient low-cost absorbents that can potentially be used in the removal of crude oil, petroleum products and toxic organic solvents. Traditional absorbents include foams and powders with high absorption capacity, fast absorption kinetics, selectivity and controllable hydrophobicity [83]. The absorption capacity of these materials is dependent on a variety of physicochemical properties such as density, surface tension, viscosity, polarity, hydrophobicity, porosity and pore size [47].

Carbonaceous materials such as activated carbon, expanded perlite, zeolites and three-dimensional (3D) porous carbon nanotube-based materials have been applied as absorbents due to their low apparent density, high porosity, large specific surface area, intrinsic surface hydrophobicity and superwettability for organic solvents and oils and environmental friendliness [84]. For instance, Gui et al. [85] developed a carbon nanotube sponge that could absorb various solvents and oils with an absorption capacity up to 180 times of their own weight.

As shown in previous sections there are a variety of processing techniques used to prepare BC-GO nanocomposite aerogels. However, due to strong interaction of GO with water, these BC-GO aerogels cannot be used for the specific separation of oily compounds. Wang et al. [86] reported a method in which BC-GO aerogels underwent further reduction in H_2_ flow leading to BC-RGO nanocomposite aerogels, which exhibited specific absorption only for organic liquids. These nanocomposite aerogels could specifically absorb 135–150 g organic liquids per g of their own weight.

Luo [56] followed a different approach. They first freeze-dried a BC-graphene pellicles in order to obtain aerogels. Then, the aerogels were carbonized at 800 °C for 2 h in a tubular furnace. The carbonized BC-graphene nanocomposite aerogel featured a honeycomb-like surface morphology and a three-dimensional (3D) interconnected porous structure. They showed high absorption capacities for a wide range of oils and organic solvents, including hexane, acetone, methanol, diesel oil, silicone oil, pump oil and soybean oil, among others. The maximum value absorption value reached was of 457 g of silicone oil per g of their own weight. This high absorption capacity is due the fact that the honeycomb-like surface provides sufficient space and active sites improving the efficiency of liquid diffusion and the hydrophobic interactions between absorbates and carbon.

On the other hand, BC-graphene membranes have been used as adsorbents for metal ions removal [35,87]. According to the Environmental Protection Agency (EPA), metal ions such as lead (Pb^2+^), zinc (Zn^2+^), cadmium (Cd^2+^), manganese (Mn ^2+^), silver (Ag^+^) and mercury (Hg^2+^) are important water pollutants from industries such as metallurgy, pharmaceutical, chemical and petroleum refining industry [88]. This represents an important threat to humans and aquatic and other life forms.

A variety of technologies have been developed to perform metal ion removal, including reverse osmosis, ultrafiltration, ion exchange, coagulation, floatation, chemical precipitation, electrodialysis, flocculation and evaporative recovery [89]. Among them, adsorption has the advantage of being essentially toxic-free, low cost, flexible and simple to operate [90]. Adsorption is a phenomenon in which pollutant metal ions associate with ions that are peripherally available on the adsorbent material.

Both, BC and graphene have been used in the development of adsorbent devices for water purification. BC has been shown to be able to receive guest molecules or inorganic particles [91,92,93]. BC nanocomposites and hybrid materials have been reported as adsorbent materials, especially for heavy metals [94,95,96,97]. Graphene oxide (GO) has oxygen-containing functional groups (i.e., hydroxyl, carbonyl, carboxyl and epoxide) on the surface, enabling them to form strong complexes with metal ions. Thus, GO can be used as an adsorbent for heavy metal ions and dyes [98]. It has been reported that electrostatic interaction is the mechanism behind the cationic heavy metal remediation; between metal cations and negative surface charge and/or electrons of GO-based materials [99].

Mensah et al. [87] prepared an adsorbent membrane by esterification of bacterial cellulose (BC) and graphene oxide (GO), containing hydroxyl, alkyl and carboxylate groups. The results showed that the BC-GO samples prepared have a specific surface area of 49.99 m^2^/g and an average pore size of 18.696 Å with a total pore volume of 0.0356 cm^3^/g. Batch experiments–adsorption studies confirmed the material to have a very high Pb^2+^ removal efficiency of over 90% at pH 6–8. The maximum adsorption was 214.3 mg/g for the 60 mg/L initial concentration of Pb(II) ions. Fang et al. [35] also prepared BC-GO membranes for ion adsorption. The BC-GO membrane prepared was shown to have selective ion permeation according to the size of ions. They found that ions of K^+^ and Cl^−^ with small size (hydrated radii < 4 Å) can quickly diffuse through the BC-GO membrane while RhB with large ion size (hydrated radius > 1 nm) cannot. This suggests that this BC-GO nanocomposite membrane could be used not only for water purification but also as a selective ion permeation device.

### 3.3. Bacterial Cellulose-Graphene Nanocomposites for Biomedical Applications

BC has been shown to be a biocompatible and non-toxic material with potential applications in the biomedical field [3,4,100]. Novel BC-based nanocomposites have been developed for a variety of biomedical applications including scaffolds for tissue engineering [101,102,103], skeletal and soft tissue grafts [104,105], wound dressing patches [106], drug delivery devices [107] and artificial blood vessels [108], among others.

Graphene has also been used for biomedical applications such as antibacterial materials [109,110], drug delivery systems [111,112] and tissue engineering scaffolds [113,114]. However, it has been reported that graphene-based nanomaterials may have toxic effects on cells and tissues. In general, nanoparticles can enter cells, if they are <100 nm in diameter. They can enter the nucleus, if they are <40 nm in diameter [115]. GO sheets can interact with cells, adhere the cell membrane and insert in the lipid bilayer. This interaction can impair cell membrane integrity and alter its functions such as the regulation of membrane associated genes, fluidity and ion channels [116].

Several factors can determine the biocompatibility of graphene-based materials, including size, surface area, surface modifications, charge and the formation of agglomerations [117,118,119]. Some studies suggest that graphene-based materials are biocompatible for a specific biomedical application [120,121,122]. Some researchers have improved the biocompatibility of graphene-based materials and prevent the inflammatory response using purified graphene oxide dispersions [123] or functionalizing GO with poly(acrylic acid), which can modify the composition of the protein corona formed on the GO surface which determines its cell membrane interaction [116]. When novel graphene-based materials are developed for biomedical applications, biocompatibility must be considered, including accurate studies of their potential toxicity effects.

Table 4 shows the biocompatibility tests performed for BC-graphene nanocomposites prepared to be used in a variety of biomedical applications. The results are not comparable because they were performed using different methodologies and cell lines. However, the authors reported fairly good biocompatibility results. The biomedical applications reported for BC-graphene nanocomposites include cell culture [45], tissue engineering [68,124,125], wound dressing [126] and drug delivery [111,127,128,129].

## 4. Conclusions

Bacterial cellulose is a biopolymer synthetized by bacteria as a coherent 3D network of cellulose nanofibres. This network has remarkable mechanical properties and has been used as matrix for the preparation of nanocomposites for a variety of different applications. Graphene nanomaterials, including pristine single graphene sheets, graphene oxide and reduced graphene oxide have been used to provide polymeric materials with novel properties such as electric conductivity, absorption and adsorption capabilities. In order to take advantage of the intrinsic properties of both, bacterial cellulose and graphene, many processing techniques have been developed to successfully incorporate graphene nanomaterials, mainly graphene oxide and reduced graphene oxide. Films, membranes, hydrogels and aerogels can be produced using a variety of processing routes. The three main applications identified for these nanocomposites were the development of novel electric conductive materials and energy storage devices, the preparation of aerogels and membranes for the removal of oil and metal ions from water and a variety of biomedical applications. The incorporation of graphene has shown to greatly improve the conductivity of BC-based materials, making BC-graphene nanocomposites suitable for the assemblage of novel flexible supercapacitors that could be used for energy storage of novel flexible electronic devices. Regarding the biomedical applications, BC-graphene nanocomposites have been used in the preparation of materials for cell culture, tissue engineering, wound dressing and drug delivery. However, there are reports showing that graphene can be toxic for cells and tissues. Further investigations should be focused on the biocompatibility of these novel BC-graphene nanocomposites for biomedical applications.

## Figures and Tables

**Table 1:** Summary of bacterial cellulose (BC) and graphene properties.

Properties	Value	Observation	References
Bacterial cellulose network			
Diameter	35–90 nm	Individual nanofibres	[22]
Length	580–960 µm	Individual nanofibres	[23]
Young’s modulus	78 GPa	Individual nanofibres	[22]
Porosity	65–75%	BC native network hydrogel	[24]
Surface area	20 m^2^/g	BC native network hydrogel	[25]
Water holding capacity	201%	BC native network hydrogel	[25]
Graphene			
Surface area	2600 m^2^/g	Theoretical prediction	[26]
Mobility	15,000 cm^2^ V^−1^ s^−1^	Room temperature	[26]
Electric conductivity	10^6^–10^7^ S m^−1^	Isolated single particle conductivity	[27]
Thermal conductivity	5.8 × 10^3^ Wm^−1^K^−1^	-	[26]
Young’s modulus	1 TPa	-	[26]

**Table 2:** Conductivity of BC-graphene nanocomposites reported by different studies.

Nanocomposite	Graphene Content (%)	Conductivity (S∙m^−1^)	References
BC-Graphene	18.4–26.8	70–80	[49]
BC-RGO	2.5–10	0.001–0.01	[69]
BC-RGO	30	12	[70]
BC-Graphene-PANI	-	170	[52]
BC-RGO-NH_4_I	30	0.013	[71]

**Table 3:** Conductivity, specific capacitance and cycling stability of flexible electrodes prepared from BC-graphene nanocomposite films.

BC-Graphene System	Conductivity (S m^−1^)	Specific Capacitance (F g^−1^)	Cycling Stability (%)	Reference
BC/GO	171	160	90.3, after 2000 cycles	[41]
BC/GE/PANI	1660	645	82.2%, after 1000 cycles	[48]
Polypyrrole/Bacterial Cellulose/Graphene	1320	556	95.2, after 5000 cycles	[36]
BC/RGO	-	216	86, after 10,000 cycles	[34]
Nitrogen-Doped Carbon Networks/Graphene/Bacterial Cellulose	-	263–318	~100, after 20,000 cycles	[77]

**Table 4:** Biocompatibility of BC-graphene based nanocomposites tested for different biomedical applications.

Type of Matrix	Type of Graphene	Biocompatibility Test	Viability (Cells)	Potential Application Assessed	Reference
BC hydrogel	Graphene oxide (GO)	Mouse fibroblast cell line (L929), CCK-8 assay	0.55–1.1 (O.D. = 450 nm †)	Drug delivery system	[129]
BC hydrogel pellets	Graphene oxide (GO)	Mouse peritoneal macrophages-RAW264.7, MTT assay	~75 × 10^4^ cells (72 h)	Drug delivery system	[44]
BC film	Reduced graphene oxide (RGO)	Human marrow mesenchymal stem cells (hMSCs)	~5.5 × 10^4^ cells (72 h)	-	[45]
BC/Hydroxyapatite porous structure	Graphene oxide (GO)	MG-63 and NIH 3T3 cells, MTT	110–120% (MG-63 cells, 24 h) 80–110% (NIH 3T3 cells, 24 h)	Tissue engineering	[124]
BC/PEDOT film	Graphene oxide (GO)	PC12 neural cells, MTT	95% (24 h)	Regenerative medicine	[51]
BC hydrogel	Graphene oxide (GO)	Human dermal fibroblast	80% (24 h)	Wound dressing	[126]

† O.D.: Optical density, absorbance.

## Abbreviations

BC	Bacterial cellulose
CNT	Carbon nanotubes
DCC	Dicy clohexyl carbodiimide
DMF	N,N-dimethyl formamide
EDLC	Electrochemical double-layer capacitor
GFN	Graphene family nanomaterials
GO	Graphene oxide
N-GOQD	Nitrogen doped graphene oxide quantum dots
PANI	Polyaniline
PEDOT	Poly-3,4-ethylenedioxythiophene
RGO	Reduced graphene oxide
TEMPO-BC	2,2,6,6-tetramethylpiperidine-1-oxyl radical-oxidized bacterial cellulose
